# Efficacy and safety of analgesic-eluting urethral catheter after urogynecological surgery: a prospective randomized controlled trial

**DOI:** 10.1038/s41598-025-30343-4

**Published:** 2025-11-27

**Authors:** Yoon-Jung Jung, Suk Woo Lee, Yongju Lee, Kookjin Huh, Jae Hoon Chung, Hwanik Kim

**Affiliations:** 1 Department of Obstetrics and Gynecology, Hallym University Sacred Heart Hospital, Hallym University College of Medicine, Anyang, Republic of Korea; 2 Department of Urology, Hallym University Sacred Heart Hospital, Hallym University College of Medicine, 22, Gwanpyeong-ro 170beon-gil, Dongan-gu, , 14068 Anyang-si, Gyeonggi-do Republic of Korea

**Keywords:** Female, Analgesic-eluting urethral catheter, Bladder discomfort, Ropivacaine, Prospective randomized controlled trial, Diseases, Medical research, Urology

## Abstract

Catheter-related bladder discomfort (CRBD) and urethral pain are frequent issues following urethral catheterization, particularly after urogynecological procedures. This study evaluates the efficacy and safety of a ropivacaine-eluting urethral catheter in reducing CRBD. Sixty female patients undergoing urogynecological surgery were randomized into three groups: Control arm, 0.5% ropivacaine 1 mL/hr (Study arm 1), and 0.5% ropivacaine 2 mL/hr (Study arm 2), with 20 patients each. CRBD incidence and severity at 24 h postoperatively were assessed. Adverse events and patient discomfort were evaluated as secondary outcomes. Baseline demographics including age, ASA score, body mass index, and surgery type were not significantly different among groups (all *p* > 0.05). CRBD incidence was 65.0% (Control arm), 36.8% (Study arm 1), and 21.1% (Study arm 2) (*p* = 0.018). CRBD severity was significantly lower in both study arms compared to control (*p* < 0.001). No significant differences in adverse events were observed among groups. Catheter-related discomfort(*p* = 0.008) and patient preference for future reuse of catheter (*p* = 0.004) differed significantly. An appropriate dose of ropivacaine eluting urethral catheter significantly reduced the incidence and severity of CRBD and the discomfort with catheter usage in patients undergoing urogynecological surgery without significant adverse events while achieving preference for future reuse of analgesic-eluting catheters.

*Registry*: The Clinical Research Information Service (CRIS), Number: KCT0010934, Registration date: 22nd August 2025. (Direct link: https://cris.nih.go.kr/cris/search/detailSearch.do?search_lang=E&focus=reset_12&search_page=L&pageSize=10&page=undefined&seq=31096&status=5&seq_group=31066).

## Introduction

Catheter-related bladder discomfort (CRBD) and urethral pain are well-known postoperative complications, particularly following urogynecological surgery. CRBD can negatively affect patient satisfaction, increase opioid consumption, and hinder recovery. Various pharmacologic approaches have been attempted, including antimuscarinics and systemic analgesics, with limited success^1,2^.

Previous studies have explored the use of ropivacaine instillation into the bladder to relieve CRBD, showing modest benefit. However, these approaches typically rely on single-dose or systemic administration, which may result in fluctuating efficacy or systemic side effects. Our previous research demonstrated the utility of ropivacaine in reducing CRBD through intravesical methods, laying the groundwork for alternative delivery strategies^3,4^. This approach was previously evaluated in a randomized controlled trial using intravesical ropivacaine instillation following urologic surgery, which demonstrated a transient reduction in CRBD symptoms.

Local continuous delivery of anesthetics may offer better symptom control with fewer systemic side effects. Ropivacaine, a long-acting amide local anesthetic, can be safely administered at low continuous doses^5,6^. The use of an analgesic-eluting catheter is a novel approach to mitigate CRBD directly at the site of irritation.

This randomized controlled trial aims to evaluate the efficacy and safety of a ropivacaine-eluting urethral catheter in reducing CRBD in patients undergoing urogynecological surgery.

## Materials and methods

### Study design and setting

This was a prospective, single-center, multidisciplinary, randomized controlled trial (RCT). The study protocol was approved by the Institutional Review Board (IRB number: HALLYM 2023-09-001–002), and written informed consent was obtained from all participants. The study adhered to the principles of the Declaration of Helsinki. The study was also registered with the Clinical Research Information Service (CRiS), Republic of Korea (22/08/2025, KCT0010934). The registration entry was finalized administratively after enrollment (1st enrollment date: 24/01/2024, Final enrollment date: 02/01/2025) had begun, but all outcomes were predefined and transparently reported. We acknowledge that this timing does not meet the ICMJE requirement for prospective trial registration. However, all study procedures—including inclusion criteria, randomization, interventions, and predefined primary and secondary endpoints—were fixed in the IRB-approved protocol prior to the initiation of enrollment, and no protocol modifications were made thereafter. The retrospective registration has been transparently disclosed, and all results have been reported according to CONSORT guidelines.

### Participants

In this study, our concept of ‘urogynecological’ surgery included both urological and gynecological surgery. The inclusion criteria were as follows^1^: Patients aged 19 to 79 years who require urinary catheterization after surgery during the study period and are scheduled to undergo catheterization (No bladder or urethral surgeries were included)^2^. Patients with an Eastern Cooperative Oncology Group performance status of 0 or 1 who have the sufficient activity level to perform normal daily activities^3^. Patients who can read and consent to the informed consent form.

Exclusion criteria were as follows^1^: Patients who have received prior treatment for surgical diseases, such as radiation therapy, chemotherapy, or hormone therapy before surgery or who are scheduled to receive such treatment after surgery^2^. Patients with a history of previous surgery or radiation therapy in the pelvic cavity^3^. Patients with allergies to analgesics^4^. Patients with severe hypertension^5^. Pregnant women^6^. Patients who do not voluntarily wish to participate in this study^7^. Patients deemed unsuitable for participation in the clinical trial at the investigator’s discretion.

### Intervention

**Fig. 1:**
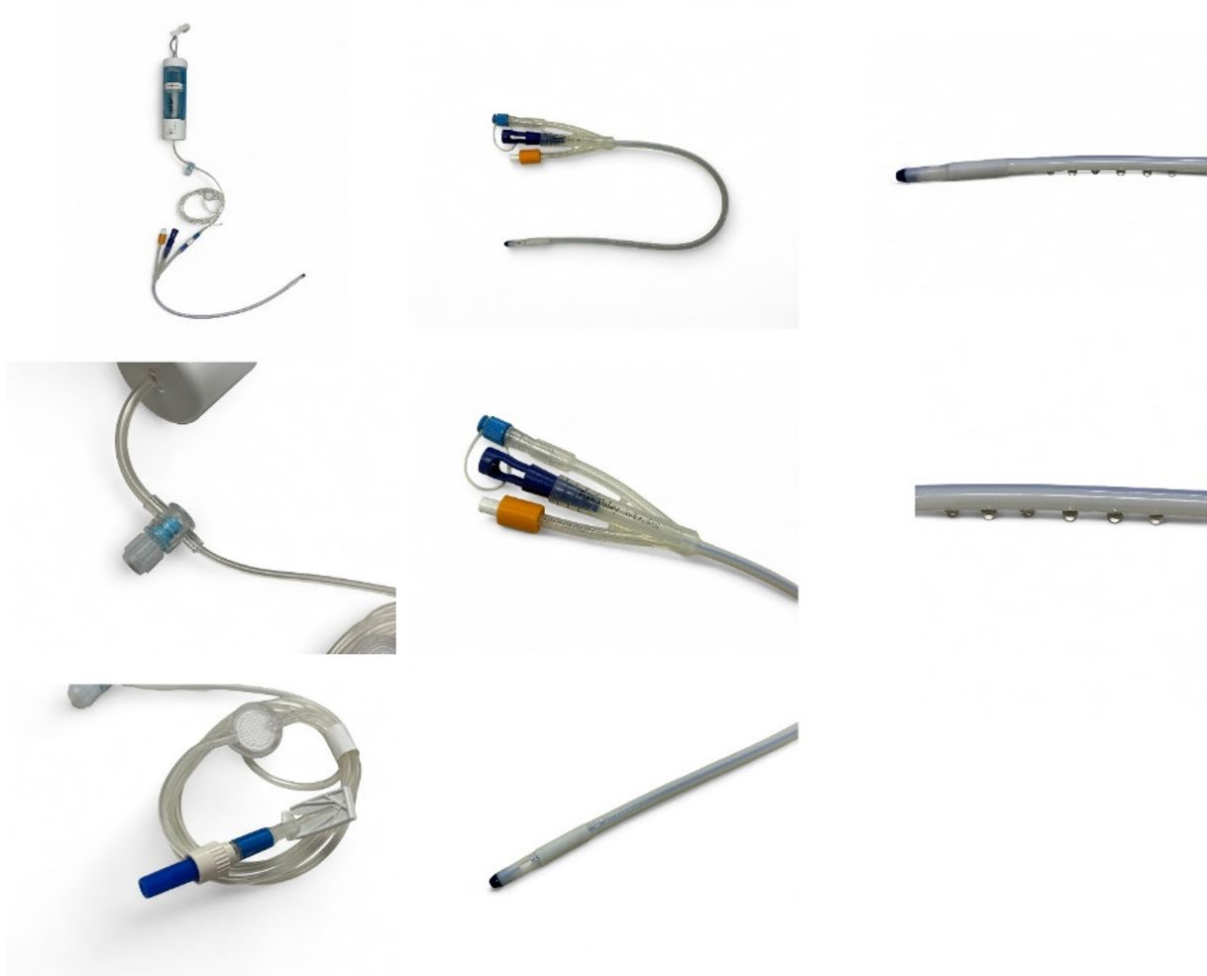
Visual representation of the FreeFoley urinary catheter.

Participants were randomized into three groups:

- Control group: FreeFoley urethral catheter (Fig. 1) with normal saline infusion (1 mL/hr) without analgesic.

- Study Arm 1: 0.5% ropivacaine delivered at 1 mL/hr via FreeFoley catheter.
- Study Arm 2: 0.5% ropivacaine delivered at 2 mL/hr via FreeFoley catheter.

The FreeFoley system is a specialized drug-eluting Foley catheter that allows for continuous infusion of analgesic directly into the urethra via multiple pores while maintaining urinary drainage. More detailed technical descriptions of the device configuration and drug delivery mechanisms have been previously reported^3^. Furthermore, in the case of the FreeFoley catheter used in this study, the number of pores is set to six and the maximum drug release length is 3 cm, considering the average urethral length in women. Drug release commences 1 cm distally from the balloon site of the Foley catheter.

### Outcomes

The primary outcomes were CRBD incidence and severity at 24 h postoperatively. The secondary outcomes were adverse events and patient-reported discomfort related overall urethral catheterization.

### Randomization and blinding

Randomization was performed using a computer-generated sequence with a 1:1:1 allocation ratio. Allocation was concealed in opaque, sealed envelopes until the time of catheter placement. Due to the nature of the intervention, blinding was not feasible for physicians; however, outcome assessors were blinded to group assignment.

### Infusion protocol

All indwelling catheters were inserted with a standardized balloon volume of 5mL, ensuring consistency across groups. The ropivacaine infusion was initiated immediately postoperatively and maintained continuously for 24 h. The catheter was connected to an elastomeric pump calibrated to deliver the pre-specified volume without external power.

### Assessment of CRBD, perioperative voiding symptom, and patient-reported discomfort

CRBD was defined as a burning sensation in the suprapubic area or an urge to void. The perioperative voiding symptoms were assessed using the International Prostate Symptom Score (IPSS) and the Overactive Bladder Symptom Score (OABSS) questionnaires. Voiding symptoms were assessed preoperatively on the day of admission without a catheter and postoperatively after completing a voiding trial following catheter removal. Patient-reported discomfort was defined as discomfort felt by subjects throughout the process of using urinary catheters postoperatively. CRBD incidence was assessed using free text question reflecting CRBD definition: ‘Is there any urge to pass urine or discomfort in the suprapubic region?’. Authors evaluated patient has CRBD if they responded positively. CRBD Severity was assessed using a numerical rating scale (0–10). The authors employed the visual analog scale (VAS) assessment tool, which presented facial expressions alongside their corresponding scores on a numerical rating scale. Subsequently, the patients were requested to document their respective scores. Regarding patient-reported discomfort, subjects were asked to respond to the following question: “What is the level of discomfort experienced with the use of postoperative analgesic-eluting urethral catheter?“. Patients were asked to indicate one of the following options; ‘Not at all’, ‘Slightly’, ‘Moderate’, ‘Very’, or ‘Extremely’.

### Evaluation of adverse events

Patients were systematically queried through interview with one of our investigators for adverse events including urinary retention, hematuria, pericatheter leakage, and systemic toxicity.

### Sample size calculation

Sample size was calculated to detect a 40% difference in CRBD incidence and severity between control and intervention arms with 80% power and α = 0.05, requiring at least 17 patients per group. Considering a 10% dropout rate, 60 patients were enrolled. The 40% difference was selected based on effect sizes reported in prior CRBD studies^7–9^. Although the enrolled population was modest in size, the trial was not designed as a pilot study.

### Statistical analysis

All statistical analyses were conducted using the IBM SPSS version 27.0 (IBM Corp., Armonk, NY, USA). Clinical characteristics of eligible subjects were compared between groups according to the analgesic drugs and their infusion rate using the chi-square test and Fisher’s exact test for categorical variables and a one-way ANOVA test for continuous variables^3,4^. In addition to ANOVA, as for VAS comparison specifically, pairwise comparison with Dunnett’s test was also performed whether there is any statistical significance between two groups (Control vs. Study 1, Control vs. Study 2). Quantitative variables were presented as mean and standard deviation, while categorical variables were reported using frequency and percentage. The last observation carry-forward method was used to address missing data if any. Statistical significance was set at *p* ≤ 0.05.

## Results

**Fig. 2:**
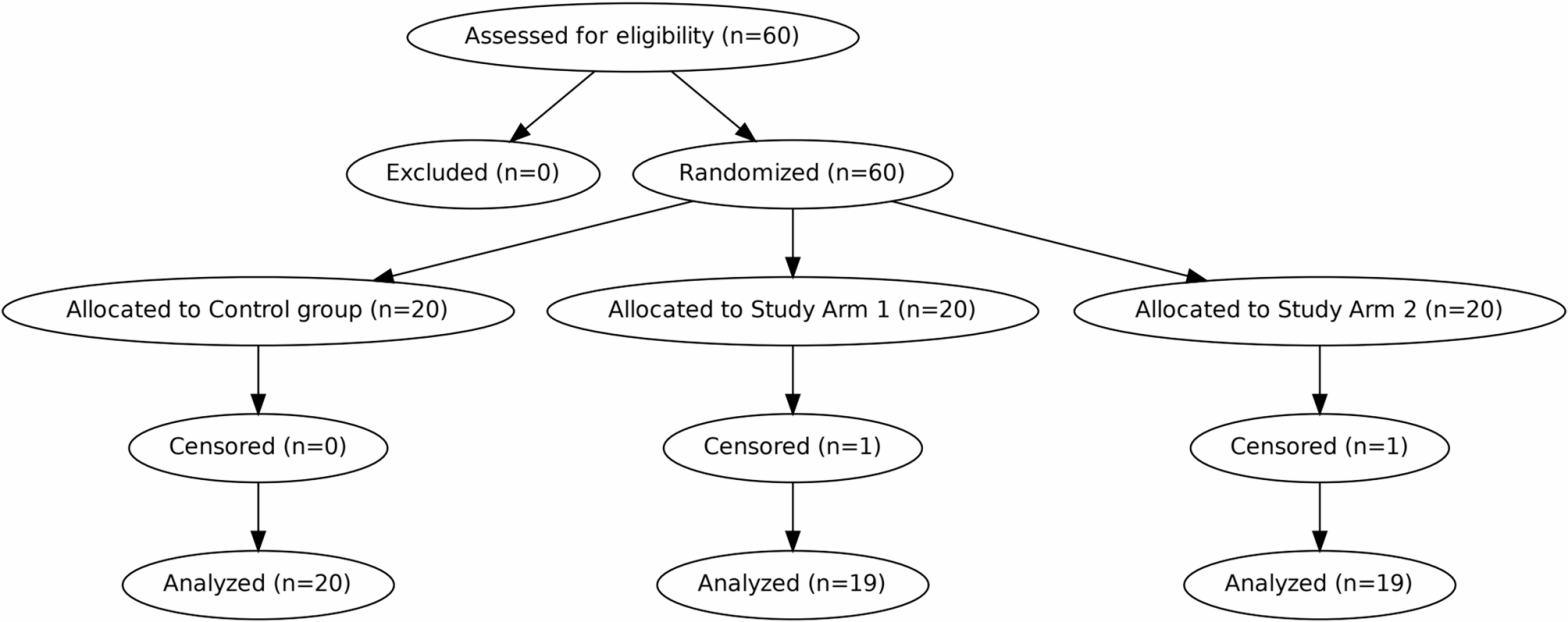
CONSORT flow diagram. Flow of participants through each stage of the randomized controlled trial, including enrollment, allocation, follow-up, and analysis.

All 60 subjects met the study criteria, and 58 were included in the final analysis assessed between 23/01/2024 and 04/01/2025 (Fig. 2). Two were censored: one patient experienced catheter blockage caused by postoperative bleeding unrelated to the catheter’s malfunction or the drug, and another patient experienced an unexpected delayed postoperative recovery, resulting in the removal of her catheter on postoperative day 7.

The subjects’ mean age was 51.7 years old. Baseline demographics including age, ASA score, body mass index, and surgery type were not significantly different among groups (all *p* > 0.05) (Table 1). The mean duration of catheterization is 1.3 ± 0.5 days, with a median of 1 day (interquartile range 1 day). These values were consistent across groups. No significant differences were observed in the pre- and postoperative IPSS and OABSS questionnaire scores among the groups (Tables 1 and 2). Specifically, the spectrum of renal surgeries included retrograde intrarenal surgery, endoscopic combined intrarenal surgery, and percutaneous nephrolithotomy for urinary stones, robotic partial nephrectomy for renal masses and robotic pyeloplasty for ureteropelvic junction obstruction (Table 1).

**Table 1:** Baseline characteristics of study participants.

	Control arm (*n* = 20)	Study arm 1 (*n* = 19)	Study arm 2 (*n* = 19)	*p* value
Age (Year)	50.8 ± 13.6	50.3 ± 15.3	54.1 ± 13.7	0.680
ASA score 1–2	20 (100.0%)	19 (100.0%)	18 (94.7%)	0.598
3	0	0	1 (5.3%)	
Body mass index	25.4 ± 5.2	25.9 ± 4.7	26.2 ± 5.1	0.878
Diabetes mellitus	0	3 (15.8%)	3 (15.8%)	0.172
Hypertension	6 (30.0%)	4 (21.1%)	5 (26.4%)	0.815
Previous catheter history	3 (20.0%)	5 (26.4%)	5 (26.4%)	0.565
Operation procedure				0.383
Surgery regarding Uterus	7 (35.0%)	10 (52.6%)	10 (52.6%)	
Surgery regarding Ovary	2 (10.0%)	4 (21.1%)	2 (10.5%)	
TLH + BSO	6 (30.0%)	1 (5.2%)	2 (10.5%)	
Renal surgery	5 (25.0%)	4 (21.1%)	5 (26.4%)	
Length of stay (Day)	3.0 ± 1.2	3.5 ± 1.9	3.6 ± 1.6	0.476
Length of postoperative catheterization (Day)	1.2 ± 0.7	1.1 ± 0.3	1.3 ± 1.0	0.197
Total IPSS score (Pre)	2.2 ± 2.5	3.9 ± 3.9	3.0 ± 3.0	0.263
IPSS QoL score (Pre)	1.9 ± 0.9	2.4 ± 1.3	2.2 ± 1.2	0.851
Total OABSS score (Pre)	1.8 ± 1.6	3.2 ± 3.7	2.1 ± 2.2	0.224

Values are presented as mean ± standard deviation or frequency (percentage).

ASA: American Society of Anestheologist, TLH: Total Laparoscopic Hysterectomy, BSO: Bilateral Salpingo-Oophorectomy, IPSS: International Prostate Symptom Score, Pre: Preoperative, QoL: Quality of Life, OABSS: Overactive Bladder Symptom Score.

**Table 2:** Postoperative outcomes at 24 h.

	Control arm (*n* = 20)	Study arm 1 (*n* = 19)	Study arm 2 (*n* = 19)	*p* value
CRBD incidence	13 (65.0%)	7 (36.8%)	4 (21.1%)	0.018
Bladder discomfort score (pre-catheterization) (VAS)	0.0 ± 0.0	0.2 ± 0.5 p value: 0.449*	0.0 ± 0.0 p value: 1.000†	0.154
Bladder discomfort score (post-catheterization) (VAS)	1.8 ± 1.7	0.5 ± 0.8 p value 0.007*	0.3 ± 0.6 p value: 0.001†	< 0.001
Complication rate	0	0	0	1.000
Pericatheter leakage	0	0	0	1.000
Acute urinary retention	0	0	0	1.000
Total IPSS score (Post)	2.9 ± 2.3	3.3 ± 3.5	2.8 ± 2.5	0.363
IPSS QoL score (Post)	2.3 ± 1.1	2.4 ± 1.2	2.1 ± 0.9	0.733
Total OABSS score (Post)	2.2 ± 1.8	2.9 ± 3.5	2.2 ± 1.9	0.577

*Control vs. Study 1; †Control vs. Study 2.

Values are presented as mean ± standard deviation or frequency (percentage).

CRBD: Catheter-Related Bladder Discomfort, VAS: Visual Analog Scale, IPSS: International Prostate Symptom Score, Post: Postoperative, QoL: Quality of Life, OABSS: Overactive Bladder Symptom Score.

**Fig. 3:**
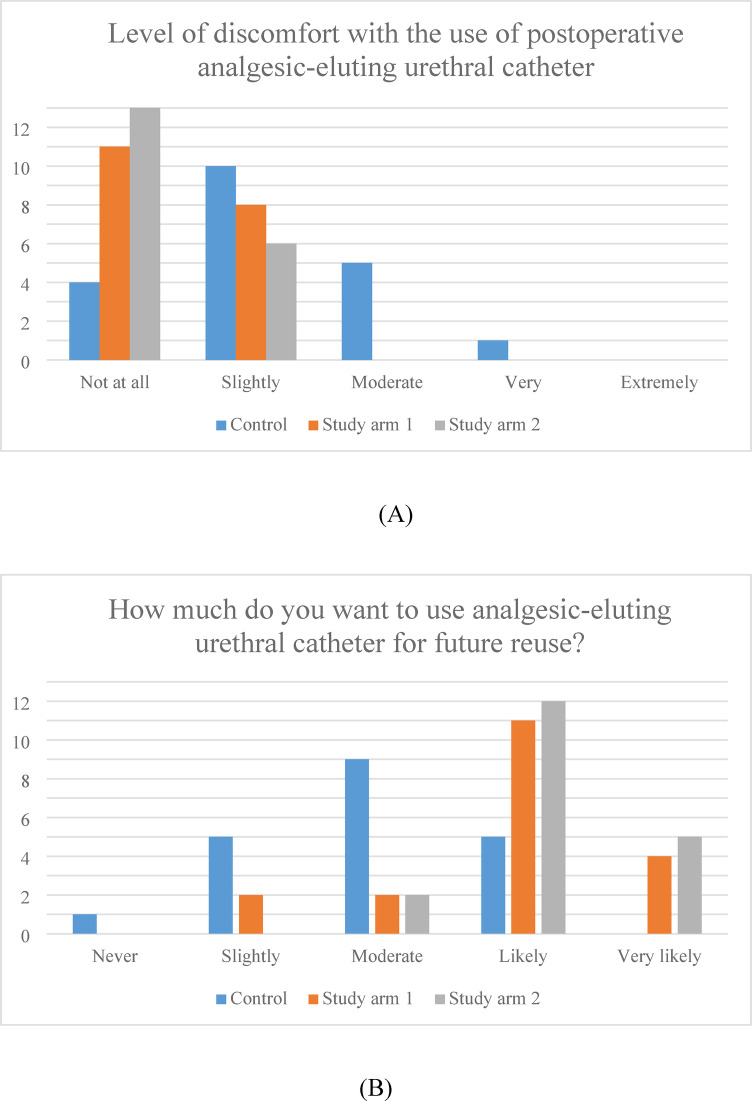
Discomfort and preference of analgesic-eluting urethral catheter use by group. (**A**) Discomfort (*p* = 0.008), (**B**) Preference (*p* = 0.004).

CRBD incidence at 24 h was 65.0% in the control arm, 36.8% in Study arm 1, and 21.1% in Study arm 2 (*p* = 0.018). Postoperative CRBD severity, evaluated as visual analog score (VAS) scale, was significantly lower in both Study Arms 1 and 2 compared to control (VAS: 1.8 ± 1.7 [Control arm] vs. 0.5 ± 0.8 [Study arm 1] vs. 0.3 ± 0.6 [Study arm 2]; *p* < 0.001). Postoperative CRBD severity also significantly differed when compared within two groups (*p* = 0.007 [Control vs. Study 1], *p* = 0.001 [Control vs. Study 2]). Patient-reported overall discomfort regarding catheter utilization differed significantly among groups (Fig. 3 (A), *p* = 0.008). Two patients in study arm 1 reported very much discomfort with the catheter usage. Preference trend for future reuse of analgesic-eluting catheter was significantly higher in both study arms (Fig. 3(B), *p* = 0.004).

No significant differences were observed in adverse events among groups. There were no case of pericatheter leakage (leakage of urine and infused drug solution), acute urinary retention or gross hematuria observed (Table 2).

## Discussion

This study demonstrates that continuous local delivery of ropivacaine via an analgesic-eluting catheter significantly reduces the severity of CRBD and improves overall discomfort in the postoperative period. Our study builds upon our prior works investigating intravesical and urethral ropivacaine delivery for the management of catheter-related discomfort. Notably, the Female-only patient population, and extended surgical indications in the current study are entirely distinct from those reported in our previous trials^3,4^. Although postoperative catheterization lasted on average less than two days in our study, CRBD remains highly prevalent, with incidence reported in 47–90% of catheterized patients even after short-term use^10^. Such discomfort, even when reflected by low VAS scores (< 2), can adversely affect postoperative recovery—leading to patient agitation, increased analgesic consumption, and delayed mobilization. A recent systematic review highlights that CRBD stems from muscarinic receptor-mediated bladder spasms and often remains inadequately managed, potentially prolonging hospitalization^8^. Thus, our intervention of ropivacaine infusion might meaningfully improve patient comfort and early recovery. These distinctions ensure that the findings in this manuscript represent a novel contribution to the literature.

Notably, the reduction in CRBD severity was dose-dependent, with the higher infusion rate yielding the best outcomes. These results are clinically relevant given the limited efficacy and systemic side effects associated with conventional analgesics used for CRBD management. The FreeFoley system provides a targeted, localized approach that minimizes systemic drug exposure. The ability to deliver a controlled dose of ropivacaine directly to target site represents a significant advancement in perioperative pain control strategies. Moreover, the simplicity of the delivery mechanism makes it feasible for routine clinical use without requiring major changes to surgical workflows. Our findings suggest that even modest infusion rates can yield substantial symptomatic improvement.

Comparing our findings to previous studies that employed systemic or intravesical analgesics^9,11–13^, the analgesic-eluting catheter appears to offer superior symptom control with fewer complications. This highlights the potential for catheter-based drug delivery systems in improving postoperative outcomes. As CRBD is often underdiagnosed and undertreated, especially in female patients, increased awareness and proactive management could substantially improve patient satisfaction and recovery. From this perspective, our study’s strength lies in our status as the first in the world to report the efficacy and safety of ropivacaine-eluting urethral catheters in a prospective RCT with an exclusively female study population undergoing urological and gynecological surgeries. Meanwhile, in our previous studies^3,4^, discomfort due to pericatheter leakage was unignorable in male. However, in this study, pericatheter leakage was relatively rare, and there was no significant difference between the groups. Nevertheless, since the number of subjects in each group was small, clinicians should be cautious when applying these results to their real-world clinical practice.

Our findings suggest that continuous local delivery of ropivacaine via an analgesic-eluting catheter effectively reduces CRBD and urethral discomfort in women following urogynecological surgery. These results align with previous reports emphasizing the influence of device-level and pharmacologic strategies for CRBD mitigation. In a prospective study of Zugail et al.^14^, the researchers demonstrated that reducing the catheter balloon volume by 50% significantly decreased both CRBD symptoms and pain scores (*p* < 0.05). This finding highlights the role of mechanical irritation in symptom pathogenesis. Another study by Hatayama et al.^12^ found that the scheduled administration of intravenous acetaminophen reduced CRBD scores at 8 h postoperatively and lowered the need for additional analgesics in patients undergoing transurethral resection of bladder tumor. As posited by Young et al.^13^, the administration of intravesical botulinum toxin has been demonstrated to provide symptomatic relief for patients with long-term catheters suffering from bladder pain and catheter bypass leakage. These findings support the implementation of a multimodal approach, which integrates device optimization, sustained local anesthesia, and systemic analgesia, to effectively manage CRBD. In our cohort, the mean VAS scores for CRBD were lower than those reported in prior studies of male patients undergoing transurethral surgery. This might reflect sex-related differences in pain perception and the less urethra-invasive nature of urogynecological procedures. Although our results were statistically robust, the clinical significance should be interpreted with caution, and future studies are required to establish the number needed to treat and the broader clinical utility of analgesic-eluting catheters in female patients. The development of innovative catheter designs may also prove to be a significant contributing factor. Drake et al. reported that advancements in catheter materials and coatings may mitigate patient discomfort and infection risk. This finding highlights the contribution of device engineering to patient outcomes.

In female cohorts undergoing gynecologic surgery, several predictors of CRBD have been consistently identified in the previous studies. In one Chinese prospective RCT^10^, age ≥ 50 years and uterus-related laparoscopic procedures were independent predictors of both the incidence and the moderate-to-severe spectrum of CRBD, whereas the absence of additional analgesics near the end of surgery increased risk; higher postoperative pain scores also tracked with CRBD severity. These observations align with a focused literature review that categorizes predictors into patient, surgical, anesthetic/analgesic, and device/insertion-technique factors, noting female-specific exposures such as obstetric/gynecologic surgery and obstetric history^16^. Contributors for CRBD could be clinically deducible in device-level: while female patients commonly receive 14–16 Fr urethral catheters, balloon position/volume appears pivotal; a prospective study^14^ demonstrated that reducing balloon volume by 50% significantly improved catheter tolerance and CRBD symptom grades, supporting routine attention to balloon inflation and trigonal contact. Taken together, these results suggest that in women—especially those undergoing uterus-related laparoscopy and older patients—anticipatory multimodal analgesia (including a terminal intra-operative dose) and device optimization (appropriate catheter size, lubrication, and conservative balloon volumes) might meaningfully mitigate CRBD and complement catheter-based local analgesic strategies as evaluated in our trial. The authors acknowledged that the distribution of surgical types, including a higher frequency of total laparoscopic hysterectomy + bilateral salpingo-oophorectomy (TLH + BSO) in the control group, might have influenced the CRBD outcomes, although there was no significant difference among the groups for all four operation types. This suggests that our results should be interpreted with caution. Meanwhile, although various data^10,16–18^ support a selective strategy focusing the analgesic-eluting catheter on patients at higher predicted risk of CRBD rather than universal use, the authors assert that the safety and efficacy of the FreeFoley catheter system have been confirmed through this study, making it suitable for use in all female patients seeking to prevent CRBD. Additionally, it is imperative that future studies be conducted to provide and validate a pragmatic risk score for the selective deployment of the FreeFoley catheter.

Despite promising results, our study has limitations. First limitation of this study is that the significance of a difference in pain or discomfort scores does not guarantee clinical significance due to the lack of previous studies. In fact, it should be noted that the number of subjects in the experimental group who reported a pain score or discomfort score of zero was not the majority, and the fact that the catheterization was inserted did not mean that pain or discomfort was present, and pain and discomfort were assessed as subjective concepts. It is believed that a larger number of subjects and a double-blind or triple-blind study would secure better objectivity and increase the reliability of clinical application. Second, it was difficult to exclude and analyze pain medication used to reduce pain from the surgery itself, which could be a confounder. The study subjects were relatively literate, so they may have been able to distinguish between pain caused by the surgery itself and pain caused by catheterization, but this study did not evaluate pain caused by the surgery separately. As detailed analgesic consumption data were not also collected, the objective measurement of systemic analgesic requirements could have strengthen our results. This should be considered in future studies. Third, the size of the catheterization tube and the number of pores through which the pain medication was injected were uniform, so they were not individually tailored to the length of each patient’s urethra. For example, some patients may have had a relatively small number of pores compared to the length of their urethra, which may have resulted in lower analgesic effect than expected. In such cases, it is expected that women may be able to achieve greater analgesia with the male Foley catheter. Meanwhile, although authors acknowledged that IPSS is validated for chronic symptoms over one month and is not well suited for acute postoperative discomfort, they endeavored to elucidate insignificant impact on voiding function after this catheter system. Nevertheless, its application in other cohorts should be interpreted with caution, and female-validated questionnaires such as International Consultation on Incontinence Questionnaire-Female Lower Urinary Tract Symptoms (ICIQ-FLUTS) may be more appropriate in future studies. Although the IPSS questionnaire was used more frequently due to our institutional practice, the ICIQ-FLUTS may have provided a more appropriate assessment of symptoms in this cohort. The reuse preference survey should also be interpreted with caution, as patients in the control group responded based on hypothetical preferences and had no direct experience with the drug-eluting catheter. Finally, the relatively small sample size may limit generalizability, and the single-center design may introduce selection bias. While the number of participants is relatively small, the trial was prospectively powered and hypothesis-driven, and therefore should not be interpreted as a pilot study. Additionally, our follow-up was restricted to 24 h postoperatively, and long-term outcomes such as late-onset urinary complications or infections were not assessed. The primary intent of this trial was to evaluate perioperative CRBD rather than long-term outcomes. Our findings suggest potential value of analgesic-eluting catheters in improving immediate postoperative comfort. Nonetheless, the generalizability of our results is limited to short-term catheterization, and future research should focus on prolonged catheter use. Though no adverse events were observed in this trial, this result should be interpreted with caution. Our sample size and the short 24-hour observation period limit the ability to draw firm conclusions regarding safety regarding not only infection but also stricture, and anesthetic toxicity. Future multicenter studies with larger populations and extended follow-up periods (ex. VAS done at 2–4 week follow-up) are needed to validate our findings and explore additional clinical applications.

## Conclusion

An appropriate dose of ropivacaine eluting urethral catheter significantly reduced the incidence and severity of CRBD and the discomfort with catheter usage in patients undergoing urogynecological surgery without significant adverse events while achieving preference for future reuse of analgesic-eluting catheters. Our exploratory evidence and techniques showed promise for routine use in the perioperative management of female patients. Nevertheless, additional multicenter study with larger population and extended follow-up will be necessary to validate and replicate the safety profile of the FreeFoley system.

## Data Availability

The datasets used and/or analyzed during the study are available from the corresponding author on reasonable request.
